# Continuous Glucose Monitor: Reclaiming Type 2 Diabetes Self-efficacy and Mitigating Disparities

**DOI:** 10.1210/jendso/bvae125

**Published:** 2024-06-27

**Authors:** Kevin Ni, Carolyn A Tampe, Kayce Sol, Lilia Cervantes, Rocio I Pereira

**Affiliations:** Medicine Service-Endocrinology, Denver Health, Denver, CO 80204, USA; Department of Medicine, University of Colorado, Aurora, CO 80045, USA; Medicine Service-Endocrinology, Denver Health, Denver, CO 80204, USA; Medicine Service-Endocrinology, Denver Health, Denver, CO 80204, USA; Department of Medicine, University of Colorado, Aurora, CO 80045, USA; Medicine Service-Endocrinology, Denver Health, Denver, CO 80204, USA; Department of Medicine, University of Colorado, Aurora, CO 80045, USA

**Keywords:** continuous glucose monitor, social determinants of health, type 2 diabetes, diabetes disparities

## Abstract

**Context:**

The rise in continuous glucose monitor (CGM) use has been characterized by widening disparities between the least and most socially marginalized. Given access barriers, there is limited CGM patient experience information that is inclusive of those with type 2 diabetes mellitus from socially marginalized backgrounds.

**Objective:**

To understand the CGM usage experience in the primary care setting across a US Medicaid population with type 2 diabetes at federally qualified health centers.

**Methods:**

This qualitative study used semi-structured phone interviews with 28 English- or Spanish-speaking participants prescribed the CGM who were enrolled in a US Medicaid program that subsidized CGMs. Audio recordings of interviews were transcribed and analyzed by reflective thematic analysis.

**Results:**

Twenty-eight participants (75% female, median age 56 years with interquartile-range 48-60 years) were interviewed. Participants were from different racial/ethnic backgrounds: 21% non-Hispanic White, 57% Hispanic, and 18% non-Hispanic Black. Participants primarily spoke English (68%) or Spanish (32%), and 53% reported 9 or fewer years of formal education. We identified 6 major themes: initial expectations and overcoming initiation barriers, convenience and ease promote daily use, increased knowledge leads to improved self-management, collaboration with provider and clinical team, improved self-reported outcomes, and barriers and burdens are generally tolerated.

**Conclusion:**

CGM use was experienced as easy to understand and viewed as a tool for diabetes self-efficacy. Expanded CGM access for socially marginalized patients with type 2 diabetes can enhance diabetes self-management to help mitigate diabetes outcome disparities.

Patients from socially marginalized backgrounds remain at highest risk for diabetes-related complications and mortality despite calls for comprehensive diabetes care [1-5]. Interventions to enhance diabetes self-management in safety-net primary care clinic settings have been proposed as a tool for addressing disparities among high-risk patients [6, 7]. While continuous glucose monitoring has enormous potential as a tool to enhance diabetes self-management, its access remains especially limited and disparate for the most socially marginalized with type 2 diabetes across the United States given restrictive eligibility criteria [8]. In fact, the increase in continuous glucose monitoring use has shown widening disparities in access, with low availability to socially marginalized populations at the highest risk for diabetes-related complications, further exacerbating disparities in diabetes [9, 10]. Recently, a regional offering of a state Medicaid program made continuous glucose monitors (CGMs) available without any restrictions and at no cost for those with diabetes with the goal of removing structural barriers to access [11]. CGMs had tremendous appeal and helped those with type 2 diabetes mellitus (T2DM) achieve treatment goals regardless of racial and ethnic background [11]. As continuous glucose monitoring becomes the standard of care for those with T2DM, there is a vital need to understand the facilitators and barriers to CGM use faced by low-income, diverse populations.

Little is known about CGM initiation and delivery for patients with T2DM as both access to continuous glucose monitoring and qualitative studies on CGM user experience have primarily been limited to people with type 1 diabetes mellitus (T1DM) and privileged, non-Medicaid cohorts [12]. While Medicare has recently (since 2023) expanded continuous glucose monitoring access by removing multiple daily insulin as a requirement and including CGM coverage for adults using once-daily insulin and those with clinically significant hypoglycemia, coverage for individuals with T2DM remains very limited [13, 14]. Patients with T2DM at federally qualified health centers (FQHCs) belonging to a metropolitan safety-net health care system have had access to CGMs without any restrictions and at no cost (previously only covered for those on 3 or more daily insulin injections) since October 2020. To better understand real-world experiences of CGM use among this racially and ethnically diverse low-income population, qualitative interviews were conducted with patients with T2DM prescribed a CGM.

## Methods

### Study Design

Semistructured interviews were performed 1-on-1 by telephone between October 2022 and July 2023 in the participant's preferred language (English or Spanish). All individuals verbally consented to the study and agreed to the publication of anonymized data prior to starting the interview. Participants received a $35 gift card as compensation for their time. The study protocol was approved by the Colorado Multiple Institutional Review Board (COMIRB #21–4867). The consolidated criteria for reporting qualitative studies 32-item checklist [15] were followed (Supplementary Document S1) [16].

### Settings and Participants

Eligible participants were patients with T2DM and ≥18 years of age who were seen at FQHCs that belong to a multiclinic urban safety-net health system with 11 primary care clinics and had access to fully subsidized Libre CGM through their regional Medicaid (a regional offering of the State Medicaid program) plan that dispensed CGM by a FQHC pharmacy. Participants had access to CGM training and support from primary care clinic nurses, clinical pharmacists, and FQHC retail pharmacists.

Participants had a primary care visit between October 2020 and June 2022 and had been prescribed and dispensed Libre CGM through June 2022. Exclusion criteria included type 1 diabetes, pregnancy during the study period, management by an endocrine specialty clinician (due to focus of study on primary care-directed prescriptions), or primary language other than English or Spanish. Participants were selected by convenience sampling and recruited over the telephone.

### Libre CGM

The FreeStyle Libre 14-day and FreeStyle Libre 2 CGM (Abbott Laboratories, Chicago, IL) available to patients during the study period are intermittently scanned CGM systems with wearable sensors (on the back upper arm) that store glucose measurements every 15 minutes. Scanning the sensor with a compatible smartphone or Libre reader device every 8 hours is required for transferring all stored glucose measurements. With the Libre 2 CGM (and not Libre 14-day) systems, the smartphone application and the reader device have built-in and enabled alarms for hyperglycemia, level 1 hypoglycemia (<70 mg/dL), and level 2 hypoglycemia (<54 mg/dL) and can receive alarm triggers from the sensors wirelessly without the need to physically scan the device.

### Interview Guide

The interview guide was developed through an extensive literature review to probe participants’ CGM user experiences (Supplementary Document S2) [16]. Prior to the interview, we asked participants quantitative questions regarding their technology use, education, duration of diabetes, and use of insulin. Answers to these questions were used to describe the group as a whole and were not paired to answers to interview questions for analyses. Interview questions included open-ended questions designed to obtain participant perspectives on checking glucose, CGM initiation/use, diabetes management, and support received [17] and some closed questions designed to focus the interview on experiences (idiographic probes) relevant to the person being interviewed (eg, “Do you check your blood sugars? How? How often?” followed by “Do you find it useful to look at your glucose numbers? If so, how? If not, why not?”) as previously described [18].

### Data Collection

Interviews were performed by C.T., K.N., and K.S (research team background, Supplementary Document S1) [16]. Deidentified interview transcripts were prepared from audio recordings. Interviews with Spanish-speaking participants were collected (K.N., K.S) and transcribed and translated to English by a certified bilingual clinician (K.N.). General questions were asked followed by an interview lasting, on average, 20 minutes. Transcripts were not returned to participants for review.

The demographic data were obtained from the electronic health record. The first CGM dispense date and date of interview was used to calculate days since first CGM dispense. Pre-CGM hemoglobin A1c (closest hemoglobin A1c to CGM start, no later than 21 days after CGM dispense) was obtained.

### Analysis

Reflexive thematic analysis was performed to identify major themes and subthemes with a systematic, 6-step approach: (1) data familiarization; (2) data coding; (3) generating initial themes; (4) reviewing and developing themes; (5) refining, defining, and naming themes; and (6) producing the report [19-23]. K.N. and R.P. independently coded all transcripts and drafted codes via an inductive approach without any preconceived theory or framework. K.S. and C.T. independently coded a subset of transcripts. K.N., C.T., K.S., L.C., and R.P. met regularly to consolidate and revise codes, themes, and subthemes as interviews were completed until a coherent set of themes was developed by consensus [20, 21, 24]. Coding was done in ATLAS.ti 23 (ATLAS.ti Scientific Software Development).

## Results

Of the 52 participants contacted, 28 were interviewed [female n = 21 (75%); male, n = 7 (25%)] with a median age of 56 years and varying levels of formal schooling (Table 1). The majority (63%) of participants used the Libre reader to obtain sensor readings and the remainder used a smartphone. Not participating was due to not being available during the proposed interview (n = 10), unable to be reached at agreed interview time (n = 4), or declined (n = 10). While most participants reported continuous CGM use, 10 participants disclosed pauses in CGM use for various reasons (Table 2). One participant had not started using CGM. Answers to quantitative questions asked during the interview were tabulated (Supplementary Table S1) [16] and were notable for 100% of participants finding it useful to look at glucose numbers. We identified 6 themes (Table 3).

**Table 1.** Participant characteristics

Characteristic	n (%)^a^
Sex	
Female	21 (75)
Male	7 (25)
Age (years)	56 (48, 60)
Race and ethnicity	
non-Hispanic White	6 (21)
Hispanic	16 (57)
non-Hispanic Black	5 (18)
non-Hispanic Asian	1 (3.6)
Language	
English	19 (68)
Spanish	9 (32)
Diabetes duration (years)	14 (8, 24)
Insulin regimen	
No	11 (41)
Once daily	8 (30)
Twice daily	3 (11)
MDI	5 (19)
Pre-CGM HbA1c	
<7	7 (25)
7-8.9	8 (29)
9-10.9	4 (14)
≥11	9 (32)
Daily smartphone use	24 (86)
Use internet	22 (79)
Years of school	
≤6	2 (7.1)
>6 and ≤9	13 (46)
>9 and ≤12	10 (36)
>12	3 (11)
Days since first CGM dispense	370 (267, 542)
Daily CGM sensor scans	4.00 (3.00, 6.00)
Interruptions to CGM use	10 (37)
Using cell phone as CGM scanner	10 (37)

All interviewed participants had tried their CGM except 1 (CGM19).

Abbreviations: CGM, continuous glucose monitor; HbA1c, hemoglobin A1c.

^*a*^n (%); median (interquartile range).

**Table 2.** Reasons for CGM use interruption

Reason for CGM use interruption	Participants (n)
Arm “needing break”	3
Lost reader	2
Needing family help	1
No reason	1
Travel	3

Abbreviations: CGM, continuous glucose monitor.

**Table 3.** Major themes and subthemes

Theme and subthemes	Illustrative quotes
1. Initial expectations and overcoming initiation barriers	
Initial expectations	**Uncertainty with new technology**: “[I was] a little scared because I didn't expect it to work that well and I didn't expect it to be reading my sugar levels that quick.” (CGM03) **Facilitated positive anticipation**: “Since the doctor was recommending it to me, that meant it was good for me to try it; and since I had already seen other persons use it, I also wanted to [try it].” (CGM23, Spanish) **Anticipating pain**: “At the beginning, I was nervous because I had to place the [sensor] on my arm. I was nervous about the little needle that you place in the arm. But it didn't hurt, it didn't hurt.” (CGM24, Spanish)
Overcoming initiation barriers	**Straightforward initiation**: “It was real easy to follow instructions. They told me to try it at home and reach out to the nurse team if I needed help.” (CGM02) **Clinical training and help**: “The first [person] who trained me was a nurse. And later [I repeated the training with] the pharmacist who was [monitoring] me when they changed my insulin … they helped me and this made it very easy.” (CGM25, Spanish) **Family help with use**: “My roommate helped me because I told her I’ve got to start wearing it to monitor my sugars. So she helped me read the instructions and what not. I was apprehensive about pushing the dial on the monitor so she did it for me and it really did not hurt. That was a good surprise.” (CGM27) **Device Spanish support**: Regarding difficulty configuring the Libre reader, “In reality no, since the instruction were in Spanish.” (CGM06, Spanish)
2. Convenience and ease promote daily use	
Benefits over fingersticks	**Easy to use**: “It's pretty easy to use and follow the instructions on it. It's an easy thing to use to monitor your blood sugars.” (CGM11) **Uninterrupted use**: “[I use it] all of the time … I put it on 2 times [per month] every [two weeks].” (CGM21, Spanish) **Convenient and quick access to glucose**: “You can just use your phone and ‘boom’ it gives you the results immediately.” (CGM14) **Minimal or no sensor discomfort**: “I don't have to think about it. I don't have to brace myself for the pain prick.” (CGM27) **Enhanced privacy and portability**: “You can put it under your arm, nobody really notices what you’re doing. It's real private.” (CGM02) **Good sensor attachment**: “It stays on, like when you take a shower. It doesn't fall off. So it really does stick really good.” (CGM12)
3. Increased knowledge leads to improved self-management	
Increased understanding and knowledge	**Promoting diabetes self-monitoring**: “The Libre is accurate. Keeps me on top. I was never able to manage my blood sugars but ever since I got the Libre it's great. I can be anywhere and check.” (CGM16) **Visualizing sugars and understanding patterns:** “I like going through the charts. It gives me an opportunity to go back and see patterns so I can trace what kinds of foods I’m eating are causing a spike.” (CGM27) **Insight into food or exercise with direct feedback**: “If I check it at a certain time, then I know—Do I have to go for a longer run or a longer walk? Or ‘Don't eat this too much,’ ‘I ate the wrong foods that day,’ ‘A little bit too much’ … It gives me a heads up.” (CGM05) **Diabetes troubleshooting**: “It's really showed me what picks up my sugars like to an extreme level. Just by it letting me know ‘Hey, this isn't working or this is working,’ ‘It's a little too high or too low.’” (CGM15)
Glucose level self-awareness	**Hyper/hypoglycemia awareness**: “… I can know if I’m going up or down. If I’m about to pass out. Sometimes I get really shaky and I can't tell if it's high or low on my own so the Libre tells me.” (CGM16) **Identifying out-of-range glucose level and taking action**: “First thing I do is grab my phone and check my sugar … if my sugar is down … I have sugar supplies right next to my bed. I deal with my sugar immediately.” (CGM07)
Motivating healthy behaviors	**Increased scanning frequency**: “I was not checking my blood sugar very much when I had to stick my finger but when I switched over to the freestyle Libre I started checking it about 9 times a day.” (CGM01) **Transformative dietary and exercise modifications**: “This little device motivates me to start to eat more healthy, to exercise more and I love it.” (CGM08, Spanish) **Freedom to try foods**: “It's given me freedom to not have to worry about food. I can eat at certain times and I know when to eat and what to eat.” (CGM02) **Catalyzing medication use**: “… it's helping me to have that daily routine where I’m staying on top of it. It's a friendly reminder to make sure I get up and take my meds.” (CGM02)
4. Collaboration with provider and clinical team	**Collaborative management**: “I think she's a pharmacist. We communicate back and forth on [EMR patient portal]. She has been very helpful and awesome. Checking on me, making sure I’m doing ok. Making sure I’m taking my insulin on a daily basis.” (CGM03) **Able to share glucose data**: “I have them set up online to be able [to see my glucose data]. I upload my results to (I think it's) Libreview, and then my doctor can check. Usually she sends me an e-mail because she's been pleased with the progress.” (CGM01) **Catalyzing medication adjustments**: “My doctor previously had me on 50 [units] of insulin and now has me on no more than 25 … it is much help to be able to … inform my doctor such that he is lowering my medications.” (CGM24, Spanish)
5. Improved self-reported outcomes	
Improved glucose and nonglucose targets	**Improved glucose**: “10 years with diabetes, I have used insulin all the time. And with my medicine, I could not lower the levels until now … My [A1c] sugar levels are a 7 right now.” (CGM22, Spanish) **Weight loss**: “I’ve lost a lot of weight, which has been an added benefit.” (CMG27)
Positive emotional effect	**Sense of increased security**: “I feel safer when I am using it … I no longer have to fear that one day my sugar will go low and I won't realize it.” (CGM24, Spanish) **Well-being**: “My experience is it's very good and everyone should have it. I feel better now. And I’ve never felt better with my diabetes.” (CGM16) **Emotional support**: “It makes me feel like someone is caring for me. Like someone is there to help me get my diabetes under control. That's been very helpful. I’m understanding now what diabetes is all about.” (CGM03)
Essential for management	**Self-confidence**: “it's been also helping me just be able to stay on top of my sugar numbers … it's helping to keep me on track with my weight loss and it helps me move around so it's helping me exercise. It's given me my life back.” (CGM02) **Reclaiming ability to manage diabetes**: “My diabetes had gotten out of control before I got my Libre monitor. And I think it's sad and I think it's wrong and it's bad practice to not give a monitor to someone until they’ve had diabetes for a long time. I wished I had it four or five years ago. What happened was I was doing my best with my diabetes but I wasn't monitoring it well and I wasn't controlling it well. I got blockage in my heart.” (CGM01)
6. Barriers and burdens are generally tolerated	
Barriers to monitoring	**Sensor discomfort**: “It's painful when you put it on, that little thing. But that goes away quick.” (CGM16) **Sensor detachment**: “You have to be really careful about smacking it on anything … It's easy to pull it off. So the sticker part really ain't great.” (CGM02) **Sensor placement challenges**: “I don't know if when I put it on [the sensor] doesn't go in correctly, doesn't stick in well, or something [happens to make it not work].” (CGM06, Spanish) **Alarm fatigue, intrusion, lack of privacy**: “The little machine is really loud when your blood sugar do go high a little bit … When I first started Libre I had the little machine and that screams crazy. And when you’re in public, it's a little bit weird, and if that thing goes off it's a little embarrassing.” (CGM11) **Smartphone incompatibility**: “My phone isn't the right type.” (CGM18) **Intentional breaks from use:** “I’ll wait to put another one on after two days and then I’ll put it back on. Just to give my arm a little break because there's a big needle in it that you stick in your arm.” (CGM13) **Unintentional breaks from use**: “Kind of continuously and on and off. There's been a couple of times I went to another state for a project for work and forgot to bring it with me.” (CGM11)
Burden of self-monitoring	**Glucose-uncertainty related anxiety**: “Just knowing where my blood sugars are. Although it worries me sometimes. Better knowing than not knowing.” (CGM12) **Frustration with hyperglycemia**: “This meter goes off … I know when I haven't been eating no sugar or nothing and it comes up high.” (CGM04) **Chore with glucose monitoring**: “Lazy to put it on.” (CGM19)

### Theme 1: Initial Expectations and Overcoming Initiation Barriers

#### Initial expectations

Participants felt a range of emotions when discussing starting CGM including uncertainty about new technology. Participants were more excited to try CGM if they had observed a friend use it or had received an enthusiastic recommendation from a provider. Participants reported anticipated discomfort with CGM sensor placement given their experience with fingerstick testing that was largely overcome with initial placement.

#### Overcoming initiation barriers

Participants shared that learning how to use a CGM for the first time, either by themselves or with assistance from family or clinic staff, was quite straightforward. While a few participants were able to set up the CGM on their own, many reported receiving training and support from clinical staff (nurses, pharmacists, and FQHC retail pharmacists) for CGM initiation: “I tried on my own twice and it didn't work. I went to the pharmacy and the pharmacist showed me” (CGM26). Participants cited clinic support for connecting their smartphones: “[Pharmacist at the clinic] helped me download the cell phone app and [set up] everything” (CGM09, Spanish).

Participants mentioned family support as crucial for starting a CGM and continued use. Participants mentioned family learning at clinic visits or asking family at home to review instructions after bringing the CGM home. Some participants reported that they continued to rely on their family members to place their sensors: “If I place [the sensor], it won't adhere well; and with her, she applies/places it very well” (CGM08, Spanish). Among Spanish-speaking Latino patients, participants particularly stressed the support of family with either CGM initiation or continued use. Libre reader support for the Spanish language facilitated its use.

### Theme 2: Convenience and Ease Promote Daily Use

Participants shared many benefits of continuous glucose monitoring over point-of-care fingerstick monitoring. Participants discussed how daily use of the CGM was straightforward and reliable. Participants reported immense relief with not having to deal with pain from fingerstick testing and how their fingers have been able to recover. The CGM enabled portability and privacy with checking glucose readings. Participants valued the ability to monitor glucose on walks and travel abroad.

### Theme 3: Increased Knowledge Leads to Improved Self-management

#### Increased understanding and knowledge

Participants reported daily glucose monitoring was helpful for understanding their diabetes. One participant said: “I review the graphics and the average [glucose] I have had the last 7 days … makes me think and [consider] what I can do to improve” (CGM06, Spanish). Participants used CGM to learn about the effects of diet and exercise on glucose levels. They found the readings helpful for making changes to their diet, lowering food portions, or adjusting their exercise regimen: “It's being able to see how certain foods affect your sugar … If I ate something and I hear that beeping and it's telling me it's high, that tells me you need to either take this out of your diet, or not eat so much” (CGM27).

#### Glucose level self-awareness

The CGM was helpful for discovering low and high glucose levels enabling participants to take corrective measures promptly: “The alarms are great. Usually when it's too low or too high then I’ll know. And I know to eat some candy if it's too low. If it's too high, I’ll drink some water” (CGM12). Participants had striking revelations of how their bodies responded to certain foods and how they could be unaware of their hypoglycemia or hyperglycemia.

#### Motivating healthy behaviors

Participants described how continuous glucose monitoring catalyzed healthy behaviors. Participants noted increased glucose checking compared to a prior glucometer. They shared that having CGM enabled them to try different foods with a sense of control: “Since I know how food affects it, I can either eat what I want to eat or modify it and not eat so much” (CGM17). Additionally, participants reported motivation for adjusting their dietary and exercise routine: “I feel more empowered with what I eat and more empowered with my diet and my exercise” (CGM01). Participants reported continuous glucose monitoring catalyzing positive medication changes such as remembering to take medications. Participants felt it motivated them to follow their clinician's recommendations. One participant said, “It's helping me stay alive because I wasn't taking care of myself without it” and “I’m not having to hear [the doctor] tell me things like … ‘you’re not taking your medicine’” (CGM16).

### Theme 4: Collaboration with Provider and Clinical Team

The glucose readings from the CGM were helpful for participants to actively engage with their clinicians regarding their diabetes care. Participants highlighted their active role in spearheading their care: “My doctor, she and I have been in constant communication” (CGM06). Participants appreciated the enhanced feedback and attention from their clinicians: “I feel safe, and my doctor has already paid more attention” (CGM24, Spanish). Participants with smartphones connected to Freestyle's Libreview online portal valued how their clinicians could remotely review their readings. Participants also reported that CGM readings helped their clinicians make medication adjustments.

### Theme 5: Improved Self-Reported Outcomes

#### Improved glucose and nonglucose targets

Participants discussed how the CGM helped them reach desired glucose targets including hemoglobin A1c. Participants also shared how the CGM assisted in obtaining nonglucose targets including weight loss: “I’ve also lost like 20 pounds with this. So it's helping me make better dietary choices and it's helping me get the energy I need” (CGM02).

#### Positive emotional effect

The CGM had a significant positive impact on participants' emotional experience of diabetes. The CGM was viewed as a life-saving tool. Participant comments reflected an understanding that hyper- and hypoglycemia can be asymptomatic. They reported feeling safer with their daily routine having their CGM: “If you’re diabetic and you don't have it, then you don't know if you’re high or low … It's a lifesaver for me working outside in the heat” (CGM20). Participants shared feeling sick pre-CGM and reported feeling better after having a CGM: “When I was not taking my insulin and not checking it on a daily basis, I just felt real crappy all day every day. Now that I’m getting [glucose] under control … I feel 10 times better” (CGM03). Participants viewed having CGM as a source of emotional support.

#### Essential for management

The CGM was noted by participants as a tool that helped them gain self-confidence to manage their diabetes. Participants shared that if they did not have their CGM, their sugars could go up again: “I think if I didn't have it, [glucose] would go back to the high levels because I wouldn't have the control” (CGM22). Participants lamented not having their CGM earlier in their diabetes disease course. Some participants attributed devastating complications such as myocardial infarction to their struggles with managing diabetes pre-CGM. Participants felt that the CGM helped them stay alive and poignantly shared how the CGM could have helped them avoid complications that followed many years of previously difficult-to-manage diabetes.

### Theme 6: Barriers and Burdens Are Generally Tolerated

#### Barriers to monitoring

Participants shared various barriers to routine or continued use that were generally tolerated. While the majority of participants felt no or minimal discomfort with having the sensor on their arm, a few participants noted transient or sustained discomfort after placement: “There's times when I can actually feel the needle in my skin and it's awkward and uncomfortable … I can still feel it for a while” (CGM17). Although many participants reported good sensor-arm attachment and not needing to replace their sensors early, some participants reported continued struggles with sensor adhesion. The loud alarms were noted to be intrusive at night for other household members or as causing embarrassment in public. While the alarms were disruptive for sleep, participants valued being alerted to hypoglycemia. Participants reported better tolerating alarms with time. Participants commented on their smartphones not being compatible or difficulties with installing the phone application.

While some participants reported uninterrupted CGM use, others noted intentional or unintentional interruptions (Table 2). A few participants reported days in which they scanned minimally, which they attributed to forgetting. A few participants reported minimal benefit of glucose monitoring with a CGM either due to burnout from reviewing the readings or feeling that results were always the same.

#### Burden of self-monitoring

Participants disclosed the burdens of diabetes monitoring. Although learning about glucose readings was potentially stressful, not knowing was more anxiety-provoking. Participants mentioned persistent hyperglycemia readings as stressful when actively making efforts to manage their glucose: “Honestly sometimes it's useful and sometimes it's annoying and aggravating. I’m trying to change my diet all the time, as much as possible” (CGM11). One participant mentioned her continued hyperglycemia was reminding her that she would need to start insulin (CGM04). Some participants found glucose checking with a CGM remained a chore.

## Discussion

Our work is the first to examine the real-world experiences of a diverse population of socially marginalized patients with T2DM starting and using a CGM in a FQHC primary care setting. This work builds upon our previous findings [11] of high effectiveness of CGM use, with an average hemoglobin A1c lowering of 1.2%, in a population of low-income racially diverse patients with T2DM within an FQHC system. Our work also expands on research seeking to address CGM access disparities in T1DM [25, 26], which has informed strategies to prepare clinic staff for supporting patients in CGM use [27]. Specifically, our findings highlight high acceptability of the CGM in a low-income, diverse patient population with T2DM and identifies personalized clinic and family support as potential strategies for overcoming health or technology literacy barriers to CGM initiation and continued use. Although the CGM was viewed as very easy to learn, participants shared how they valued the training they received from clinic nurses, pharmacists, and/or FQHC retail pharmacists for CGM initiation. Having available personalized support is critical for successful CGM delivery in socially marginalized communities given barriers to health and technology literacy [6]. Of note, initial CGM sensor placement was a significant barrier for some participants, a few of whom continued to rely on family members to assist with sensor placement. Persistent family support was more frequently cited by Spanish-speaking participants, which highlights how family support is invaluable to many Latino immigrants [28].

Prior qualitative studies of socially marginalized patients with T1DM have highlighted various individual-, clinician-, and system-level barriers to CGM initiation and use, notably lack of insurance coverage and biased provider prescriptive behavior [25, 26]. In contrast, participants in our study, all of whom had insurance coverage for the CGM, reported enthusiastic clinician support for trying the CGM. Additionally, participants reported getting more positive reinforcement and support from their clinician after starting CGM use. We speculate full subsidies and CGM availability from FQHC pharmacy overcame significant clinician-level and system-level barriers. Given that documentation requirements and insurance authorization burdens pose a formidable barrier to CGM prescription in many clinics [27], our work highlights the benefit of a nondurable medical equipment mechanism of CGM delivery with the additional benefit of engaging FQHC retail pharmacists for CGM support.

Health/numerical literacy has been linked to CGM self-efficacy and use [29], which highlights the need to identify strategies that most effectively support CGM use for catalyzing diabetes self-management in underresourced populations. Our results expand on 2 small acceptability trials of delivering CGMs to people with T2DM and socially marginalized backgrounds recruited from community settings [30, 31] highlighting how personalized clinic support can potentially support CGM use in a Medicaid cohort with a wide range of educational backgrounds. These prior studies explored promotion of self-management behaviors via diabetes self-management education (DSME) combined with CGM use: 2 DSME sessions and CGM training session prior to CGM use in African American women [30] or culturally and language-congruent (Hispanic and Spanish) peer facilitators with CGM experience providing concurrent support via an online peer support community [31, 32]. While focused on different communities, these studies highlight the potential of different strategies for providing CGM training, DSME, and CGM support/troubleshooting to enhance CGM user experience. In contrast to systematically enrolling all patients in a potentially CGM-enhancing intervention, our study examined individualized participant experiences within a system of real-world delivery of the CGM in FQHC primary care. Participants' positive experiences using different types of clinic support highlight the potential benefits of access to CGM support and resources (clinic pharmacist/nurses, FQHC retail pharmacist), which likely contributed to the broad CGM appeal and interest among those with T2DM in our Medicaid cohort regardless of race and ethnic background [11]. There is much interest and need for randomized controlled trials studying interventions to enhance the delivery of CGMs in primary care settings to improve diabetes self-management, especially among marginalized patient populations with the most to benefit. In fact, 1 ongoing current randomized control trial is examining the synergy of combining DSME with CGM use vs DSME in FQHC primary care settings [33].

Prior work in adults with T1DM identified the burden of wearing a CGM as well as how CGM use can exacerbate the burden of living with diabetes [34]. Our work extends knowledge regarding the nuanced balance of the emotional costs and benefits of diabetes monitoring in people with T2DM. While participants readily identified alarms as intrusive and a nuisance, they also reported improved tolerance of them over time. Participants also reported the importance of being alerted prior to blood glucose becoming dangerously low and the emotional benefit of knowing that the alarm would sound if their blood glucose dropped overnight. We speculate that participants were able to adjust to and tolerate alarms and wearing CGMs continuously because it provided them with a tool for regaining control of their diabetes and gaining back agency in their lives rather than seeing the CGM as controlling their lives. Having the CGM as a tool allowed participants to become self-advocates for their own diabetes care, identifying why they were not feeling well, taking corrective measures, and feeling better afterward thanks to their efforts. Of note, these benefits were reported even by participants who were not on insulin, a group often not prescribed continuous glucose monitoring tools [35].

Among people with T1DM, Messer [34] presented evidence that the CGM empowered users with a sense of security, personal control, and independence that helped promote CGM satisfaction. Similarly, in people with T2DM, our participants were proud of their own achievements. They celebrated their own transformative dietary and lifestyle modifications, which they credited to CGM-delivered insights on eating behaviors and exercise. Our findings build on prior mixed-methods work in those with T2DM suggesting quantifiable decreases in emotional burden and increases in confidence with diabetes management due to CGM use [36] by identifying a range of positive behaviors and motivations promoted by the CGM. What is striking is that for most participants the continuous feedback and learning was not viewed as a nuisance but rather as a positive motivator: “This little device motivates me to start to eat a healthier diet, to exercise more and I love it” (CGM08, Spanish). Most importantly, despite very different levels of education and health literacy, participants gained an understanding of how diet and exercise influenced the rise and fall of their blood glucose. Our findings specifically suggest the role of the CGM as a patient-centered tool for diabetes self-efficacy among diverse, socially marginalized patients.

Interestingly, the majority (63%) of participants interviewed used the Libre reader despite having smartphones. Participants reported their smartphones as not compatible or noted challenges installing the smartphone application. Thus, the shift to reader-less, smartphone-only support with newer CGMs may pose challenges for socially marginalized populations. Libre reader's Spanish-language support, which is not available with all CGM readers, facilitated its use.

Patients from socially marginalized backgrounds deal with various social barriers (ie, poverty, violence, discrimination, food insecurity) that compound and exacerbate stress and cardiometabolic health [37-39]. Toward the goal of mitigating disparities in T2DM outcomes, our prior work highlighted how fully subsidized CGMs in a Medicaid cohort was well received by patients and led to improved hemoglobin A1c across all major racial and ethnic groups and for both English and Spanish speakers alike [11]. Building on these findings, our qualitative findings highlight how the CGM was experienced by participants with T2DM in this Medicaid cohort and identified factors that made the CGM an effective tool for patients to manage diabetes despite the barriers posed by social determinants of health. Altogether, our results lend support for statewide Medicaid policymakers to expand Medicaid coverage of CGMs for people with T2DM on once-daily insulin per American Diabetes Association guidelines [14, 40, 41] especially given that such an access expansion is projected to be highly cost-effective [42, 43]. There is increasing randomized control trial data that CGMs are of benefit for people with T2DM not on insulin [14, 44], and further studies on long-term clinical outcomes (hospitalizations, emergency visits, diabetes-related complications, etc.) and cost-benefit analysis in socially minoritized and marginalized patient populations are needed to guide additional access expansion to those not on insulin to address disparities in diabetes outcomes.

### Limitations

Several limitations of our study are important to note. First, our participants did not have to deal with common insurance and delivery barriers (lengthy authorization process, ordering, and delivery coordination) [27] given the Libre CGM was on formulary, fully subsidized, and available from participants' FQHC pharmacies. We speculate that the experience of patients facing these barriers would be less positive. Second, the CGM training and support available to our participants may not be available to patients in other healthcare environments. Third, given the convenience sampling, there is a possibility that liking the CGM increased one's interest and likelihood to participate in the study. Nonetheless, interviews were continued until a coherent and rich set of themes [21] were obtained, incorporating both positive and negative experiences with the CGM. Fourth, our study was limited to individuals who spoke English or Spanish. It remains unclear how patients who do not speak English or Spanish have navigated the lack of reader/smartphone language support. Fifth, since our study was not designed or powered to be a quantitative study, the tabulations of CGM use in Supplementary Table S1 [16] were not meant to be interpreted as representative of our Medicaid cohort. Of note, some (18%) participants interviewed were no longer using a CGM at the time of interview (Supplementary Table S1) [16], and reasons for CGM use interruption since first CGM prescription were variable (Table 2), potentially limiting the generalizability of the findings.

Future studies are needed to quantify and understand CGM usage patterns and barriers to persistent CGM use especially given that socially marginalized patient populations have been noted to have less optimal CGM use [45]. There is a need for long-term studies of CGM use in those with T2DM on basal-only and no-insulin regimens characterizing the longitudinal outcomes of potential reductions in hospitalizations and macro- and microvascular complications to help guide cost-benefit analysis for access expansion. Of note, CGM use in people with T2DM on intensive multiple daily insulin regimens significantly reduced the number of all hospitalizations as well as hyperglycemia admissions within the Veterans Administration healthcare system [46]. Additionally, there is a need for studies characterizing whether intermittent CGM use can lead to sustained improvements in diabetes self-management that persist despite stopping CGM use. Interestingly, the MOBILE randomized control trial completed in a racial/ethnically and socioeconomically diverse patient population with T2DM on basal insulin showed that 6 months after discontinuing continuous glucose monitoring, its benefit on glycemic outcomes including time-in-range was largely lost [14]. Participants in our study shared fears of losing glycemic control again if continuous glucose monitoring were stopped (theme 5), and we speculate that stopping the direct continuous feedback from easily accessible glucose numbers and hyperglycemic/hypoglycemic alarms would result in eventual regression of the beneficial effects continuous glucose monitoring had on patients' daily diabetes self-management behaviors.

## Conclusion

In summary, primary care-directed continuous glucose monitoring in safety-net FQHCs is uniquely positioned to help mitigate health disparities in diabetes outcomes in low-income, socially marginalized patients who are at highest risk for life-destroying diabetes complications. The CGM was easy to understand and use and was overwhelmingly well-received by participants with T2DM from diverse backgrounds. Participants viewed the CGM as a tool for diabetes self-efficacy and became self-advocates for their diabetes care. Personalized clinic and family support helped mitigate access barriers. Expanded Medicaid and Medicare coverage for CGMs in people with T2DM has tremendous potential to help address disparities in diabetes outcomes.

## Data Availability

Restrictions apply to the availability of some or all data generated or analyzed during this study to preserve participant confidentiality or because they were used under license. The corresponding author will on request detail the restrictions and any conditions under which access to some data may be provided.

## Abbreviations

CGM: continuous glucose monitor
DSME: diabetes self-management education
FQHC: federally qualified health center
T1DM: type 1 diabetes mellitus
T2DM: type 2 diabetes mellitus
